# Effects of swimming on bone loss and mechanisms in ovariectomized osteoporotic rats

**DOI:** 10.1515/med-2026-1382

**Published:** 2026-02-23

**Authors:** Yingying Zhao, Yinyu Chen, Xinyan Yang, Yilin Wang, Yangyang Zhang, Lin Li, Peng Zhang

**Affiliations:** Department of Physical Education, Hainan Medical University, Haikou, China; Hainan Academy of Medical Sciences, Hainan Medical University, Haikou, Hainan, China; Department of Forensic Medicine & Key Laboratory of Tropical Translational Medicine of Ministry of Education, College of Basic Medical Sciences, Hainan Medical University, Haikou, China; UWE College of Hainan Medical University, Haikou, China; College of Physical Education, Huanghe S & T University, Zhengzhou, China

**Keywords:** swimming, osteoporosis, osteoprotegerin, RANK, exercise therapy

## Abstract

**Objectives:**

To investigate the effects of swimming on bone loss and the OPG/RANKL/RANK pathway in ovariectomized (OVX) osteoporotic rats.

**Methods:**

Twenty female SD rats were randomly divided into a sham-operated group, a ovariectomized osteoporosis group, a short-duration exercise group, and a long-duration exercise group. Bone volume fraction (BV/TV), trabecular thickness (Tb.Th), bone mineral density (BMD), trabecular separation (Tb.Sp), and trabecular number (Tb.N) of bone tissue were measured by Micro-CT. The expression of human C-terminal peptide collagen type I (CTX-Ⅰ), tartrate-resistant acid phosphatase 5b (TRAP-5b), bone-specific alkaline phosphatase (BALP) and serum bone glycoprotein (BGP) were measured by ELISA. The mRNA and protein expression of OPG, RANKL, and RANK in bone tissue were detected by qRT-PCR and Western bolt, respectively.

**Results:**

After swimming treatment, compared with the ovariectomized osteoporosis group, BV/TV, BMD, Tb.Th, and Tb.N increased, while Tb.Sp decreased; CTX-Ⅰ, TRAP-5b, BGP, and BALP levels decreased. OPG mRNA and protein expression increased, while RANKL and RANK decreased.

**Conclusions:**

Swimming training alleviated ovariectomy-induced bone loss and was accompanied by a shift of the OPG/RANKL/RANK axis toward an anti-resorptive profile, suggesting that modulation of this pathway may contribute to the bone-protective effects of swimming. Further functional studies are warranted to test causality.

## Introduction

Osteoporosis is commonly seen as a systemic metabolic bone disease, characterized by a reduction in bone mass and degradation of bone microarchitecture, which contributes to increased bone fragility and susceptibility to pathological fractures [1]. The main causes of osteoporosis are endocrine or nutritional factors, age, and a large number of chronic systemic diseases [2], 3]. Studies have shown that osteoporosis affects more than 200 million women worldwide [4]. It can occur at any age, particularly affecting postmenopausal women who are especially susceptible to osteoporosis, with its severe sequelae being disproportionately high, such as osteoporotic fractures [5]. It is estimated that nearly one in three women over the age of 50 will experience an osteoporotic fracture [6]. Postmenopausal women suffer from osteoporosis mainly due to a decline in gonadal function combined with aging, which promotes postmenopausal osteoporosis (PMO). The prevalence of osteoporosis in Chinese women aged 40 years and older is four to five times that of men, with 20.6 % of women and 5.0 % of men affected [7]. Women with PMO are prone to fractures, skeletal deformities and other comorbidities that seriously affect their health and quality of life, even shortening their life expectancy, and placing a financial burden on society and families [7], 8].

Currently, the main treatments for PMO include hormone replacement therapy (HRT), bisphosphonates, selective estrogen receptor modulators, receptor activator of nuclear factor kappa-B ligand (RANKL) inhibitors such as denosumab, calcium and vitamin D supplements, and probiotics. However, these approaches carry risks of side effects, compliance challenges and cost issues [9], [10], [11], [12]. Therefore, the search for new and more effective options for the prevention and treatment of PMO to provide safer, more effective and personalised treatment options is a challenge that clinical medicine and health management experts urgently need to address.

Exercise is a crucial way to increase bone formation since it may stimulate osteoblast differentiation and osteogenic capability as well as increase the metabolism of bone production [13], 14]. Swimming is an important exercise, which suitable for all ages participate and quickly gaining popularity with the general public. Previous studies suggested that swimming as an aerobic exercise can effectively improve cardiorespiratory function, lower blood cholesterol levels, increase the body’s antioxidant capacity, and slow the aging process [15], 16]. Swimming also works the muscles of the whole body and improves bone density. When exercising in water, the resistance of the water provides a complete workout for the muscles and swimming puts less stress on the bones, helping to maintain bone density [15]. Research by Orwoll et al. demonstrated that regular swimming can help older male individuals gain more bone mass, and three years older males gains more bone mineral density than the male from control group [17]. As a low-impact, full-body exercise, swimming has been widely used as an aid in the treatment of a wide range of chronic diseases such as respiratory diseases, cardiovascular diseases, obesity, arthritis and neurasthenia. It helps to improve the physical and mental health of patients by strengthening the respiratory muscles, relieving the heart, increasing energy expenditure, reducing joint stress, relaxing muscles, improving mood, enhancing memory and antioxidant capacity, among other benefits [16], [18], [19], [20].

A study has demonstrated that swimming is a low-impact exercise that exerts minimal pressure on the joints and has the capacity to effectively mitigate joint discomfort, rendering it particularly well-suited for patients afflicted with arthritis and osteoporosis [21]. As demonstrated by Figard et al. swimming has been shown to positively influence bone metabolism by increasing plasma and bone calcium levels, improving calcium balance, and enhancing net calcium absorption [22]. Meanwhile, Wochna et al. have indicated that aquatic exercise regimens result in advantageous alterations in hip and spine areal bone density, along with serum osteocalcin (OC) and C-terminal telopeptide of type I collagen (CTX) concentrations in postmenopausal women [23]. These findings suggest that swimming may constitute a beneficial form of physical activity for this demographic. However, due to the lack of direct mechanical loading on bones, swimming may be less effective than weight-bearing exercises in improving bone density. In contrast, weight-bearing exercises apply gravitational pressure to bones, more directly stimulating bone formation, but they also impose greater impact on joints [24]. Therefore, for individuals with poor joint function or severe osteoporosis, swimming, with its low-impact and joint-friendly nature, may be a safer and more suitable option.

In addition, researches discovered that the osteoprotegerin (OPG)/RANKL/RANK signaling pathway plays an important role in the regulation of bone metabolism, mainly by influencing the differentiation and maturation of osteoblasts and thus the process of bone metabolism [25]. OPG functions as a decoy receptor for RANKL, thereby impeding the binding of RANKL to RANK. This results in a reduction in osteoclast differentiation and bone resorption [26]. In osteoporosis, an imbalance in this pathway is usually the result of hyperactivity of RANKL and reduced protection by OPG, leading to bone resorption exceeding bone formation and accelerated bone loss [27]. Studies have shown that long-term fluoride exposure increases RANKL and RANK expression, leading to excessive bone resorption, while moderate exercise reduces RANKL/RANK expression and maintains OPG levels, thereby inhibiting osteoclast activity and alleviating bone resorption [28]. Additionally, exercise mitigates oxidative stress and improves the bone microenvironment, indirectly supporting the balance of the OPG/RANKL/RANK pathway, thus reducing skeletal damage caused by fluoride toxicity and exerting a bone-protective effect [28]. Furthermore, research by Pezhman et al. found that swimming training modulates the OPG/RANKL/RANK pathway, reversing the increased OPG and decreased RANKL levels in the bone and serum of type 2 diabetic rats, restoring bone remodeling balance, and improving diabetes-related bone metabolic disorders, thereby providing a protective effect on bone health [29].

However, whether swimming training can systematically alleviate osteoporosis-related bone loss and improve bone remodeling through regulation of the OPG/RANKL/RANK pathway remains unclear. We therefore hypothesized that swimming training would attenuate ovariectomy-induced bone loss by restoring the balance of the OPG/RANKL/RANK signaling axis and reducing osteoclast-mediated bone resorption. Accordingly, this study aimed to determine whether swimming training improves bone mass and microarchitecture in ovariectomized (OVX) osteoporotic rats and to elucidate whether these effects are mediated through modulation of the OPG/RANKL/RANK pathway.

## Materials and methods

### Animal subjects and ethical statement

Twenty 12-week-old (initial body weight 233.0 ± 28.3 g) female Sprague-Dawley rats were obtained from the Animal Centre of Hainan Medical University (Haikou, China). The rats were kept under standard laboratory conditions with a 12 h light/dark cycle, temperatures maintained between 21 and 24 °C, and relative humidity ranging from 40 to 70 %. Before the commencement of the experiment, the animals underwent a one-week period of acclimatization to their surroundings. All rats were fed and watered *ad libitum*. In order to guarantee the experiment’s reliability, the rats were weighed prior to grouping. This ensured that there was no significant difference in the initial weight of the rats in each group. Then, the rats were randomly divided into 4 groups (n=5 in each group): sham-operated group, ovariectomized osteoporotic group, short-duration exercise group, and long-duration exercise group. Rats in each group were anesthetized by intraperitoneal injection (3 % pentobarbital sodium by 1 mL/kg intraperitoneal injection). The devitalized rat model was established in accordance with the protocol outlined in the previous study [30]. Briefly, the abdominal cavity was entered through bilateral lumbar dorsal incisions, and the ovaries were removed bilaterally. The experimental manipulation and data collection process involved the use of blinding to mitigate the impact of subjective bias on the outcomes.

### Swimming training

The rats of exercise groups were acclimatized for one week and then trained to swim. The two exercise groups were divided into 30 min (short-duration) and 90 min (long-duration) groups according to the duration of swimming. The rats were trained once a day for eight weeks, with five times a week, and all training sessions were performed at a consistent time of day. Swimming was conducted in a tank with water temperature maintained at 32 ± 1 °C throughout the training period. During each training session, rats in the sham-operated and ovariectomized osteoporotic groups were handled similarly and placed in water at the same temperature for the same duration, but were not subjected to swimming (shallow-water exposure to avoid forced exercise) [31].

### Histopathological examination

Two femurs were randomly selected from each group, embedded in paraffin, and sectioned into 5 μm-thick paraffin slices. Hematoxylin-eosin (HE) staining was performed, and histopathological changes were observed under an optical microscope.

### Detection of bone testing indicators

The bone strength of rats was determined by the bone density and bone microstructure using micro-computed tomography (Micro-CT). Briefly, the right femur of rats were removed and preserved in 10 % formalin liquid and subsequently flattened using Micro-CT. A 3D rendering of the 2D images was generated using the 3D Creator software (Sky Scan) provided by Micro-CT. The following 3D parameters were automatically calculated using the software, including bone volume fraction (BV/TV), trabecular thickness (Tb.Th), trabecular number (Tb.N), Bone Mineral density (BMD), and trabecular separation (Tb.Sp) [32].

### Total RNA extraction, reverse transcription, and fluorescence quantification

The total RNA was extracted from the bone tissue using TRIzol. The extracted total RNA was first removed from the genomic DNA and then reverse transcribed, and the above steps were performed according to the product instructions. The cDNA was amplified according to the SYBR-Green PCR kit instructions (Takara Bio Inc. Beijing, China) and the relative expression of mRNA was calculated using the 2^−ΔΔct^ method.

### Western blot analysis

Bone tissue was ground with liquid nitrogen and lysed with lysis buffer at 4 °C and centrifuged at 14,000 r/min for 15 min to form concentrated proteins. Protein concentration was then measured using a Bradford assay. 20 μg of total protein was separated by 12 % sodium dodecyl sulfate-polyacrylamide gel electrophoresis (SDS-PAGE) for 2 h. The proteins were then transferred to a PVDF membrane by semi-dry blotting (Bio-Rad) at 90 V with carbonate transfer buffer. The membranes were closed with 5 % bovine serum albumin for 1 h at 37 °C and then incubated overnight at 4 °C with primary polyclonal antibodies, including antibodies against OPG (1:1,000; Abcam Inc., Cambridge, U.K.), RANKL (1:1,000; Beyotime Inc., Shanghai, China), RANK (1:1,000; Abcam Inc., Cambridge, U.K.), and β-actin (loading control). The membranes were then washed with TTBS in post buffer (Tris-buffered saline containing Tween-20) and incubated for 1 h at room temperature with secondary antibodies (1:5,000; Bioworld Technology, Inc., Louis Park, USA). An ECL detection system (Amersham Biosciences) was used for visualization and protein quantification was performed using an ImageQuant image analysis system (Storm Optical Scanner, Molecular Dynamics). Experiments were performed in triplicate, and band intensities were normalized to β-actin as the loading control.

### ELISA assay

The expression of human C-terminal peptide collagen type I (CTX-Ⅰ), tartrate-resistant acid phosphatase 5b (TRAP-5b), bone-specific alkaline phosphatase (BALP) and serum bone glycoprotein (BGP) in serum was measured directly using available commercial kits (Nanjing Jiancheng Institute of Biological Sciences, Nanjin, China) according to the manufacturer’s instructions. Optical density was measured using a BIO-RAD enzyme marker (Bio-Rad, Hercules, CA, USA).

### Statistics analysis

All the data were examined utilizing SPSS 22.0 statistical software (IBM, Armonk, NY, USA). Test results were expressed using the mean ± standard deviation. Differences between groups were tested using one-way ANOVA. p<0.05 was considered statistically significant.

### Compliance with ethical standards

The present work complies with the guidelines for animal studies and was conducted ethically in accordance with the Guide for the Care and Use of Laboratory, and approved by the Ethics Committee (HYLL-2023-066).

## Results

### Histopathological examination of femurs

**Figure 1:**
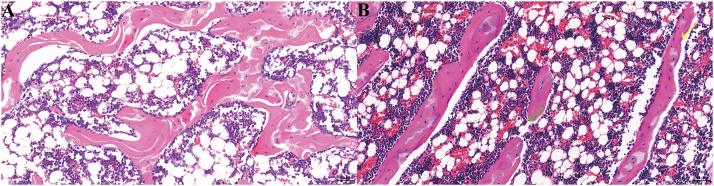
Histological observation of femoral tissue by HE staining. (A) Control group; (B) experimental group.

Compared with the control group, the femoral tissue exhibited decreased trabecular bone quantity with relatively sparse arrangement. Some trabeculae appeared thinner, and a small number showed fractures. The trabecular surfaces were lined with flattened osteoblasts. The bone marrow cavity contained abundant hematopoietic cells, with no significant inflammatory cell infiltration observed (Figure 1).

### Effect of swimming on changes in bone microstructure in ovariectomized osteoporotic rats

**Figure 2:**
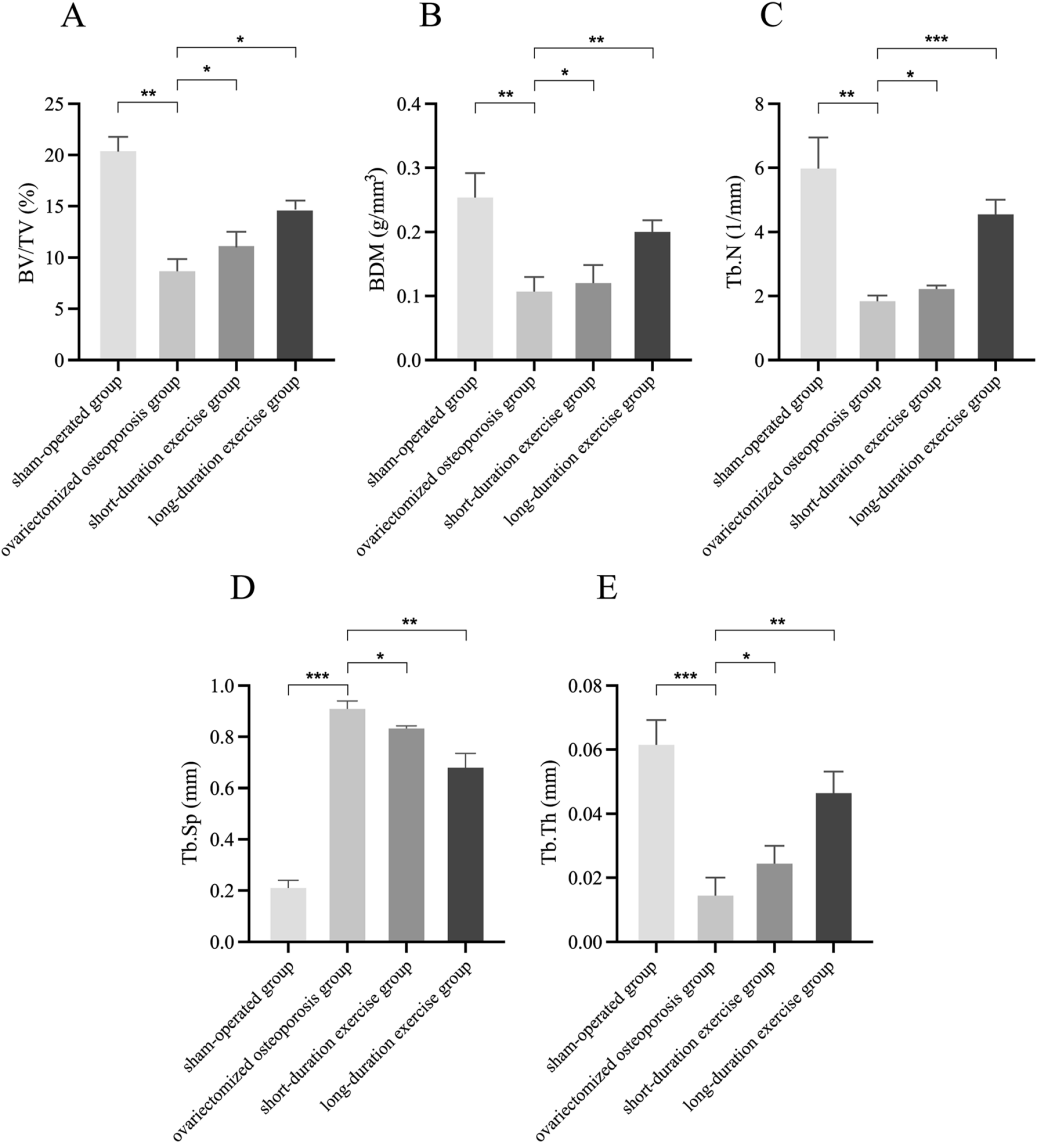
Detection of osteoporosis markers. (A) BV/TV, (B) BMD, (C) Tb.N, (D) Tb.Sp, (E) Tb.Th. Compared to the ovariectomized osteoporotic group, *p<0.05, **p<0.01, ***p<0.001.

**Figure 3:**
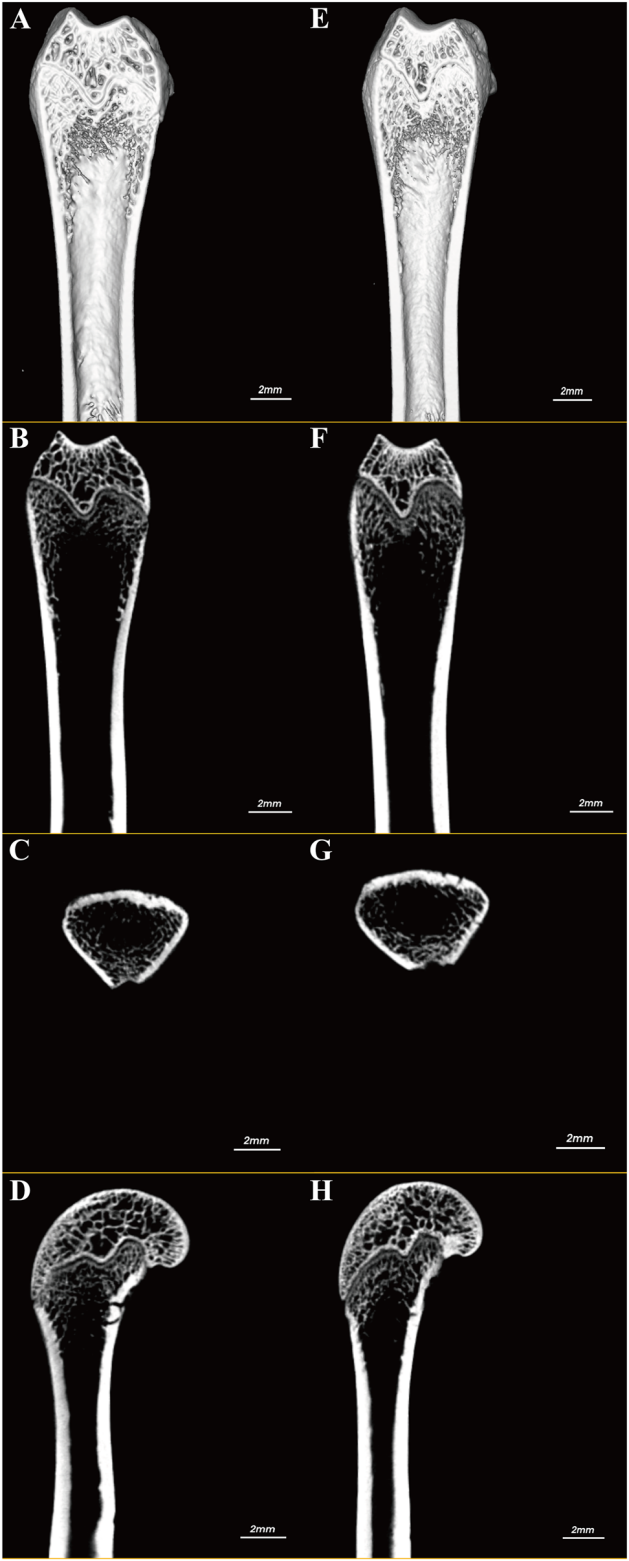
Micro-CT imaging of femoral tissue. (A–D) Control group; (E–H) experimental group. Specifically: (A) and (E) show 3D reconstructed sections; (B) and (F) display coronal views; (C) and (G) present transverse sections; (D) and (H) show sagittal sections.

In comparison with the sham operation control group, BV/TV (from 20.33 to 8.67 %), BMD (from 0.257 to 0.11 g/mm^3^), Tb.Th (from 0.062 to 0.015 mm), and Tb.N (from 6.033 to 1.897 mm) levels were decreased in the ovariectomized osteoporotic group. Meanwhile, Tb.Sp (from 0.217 to 0.917 mm) levels increased, and the difference was statistically significant (p<0.01). Following short-duration swimming training, there was a marked improvement in the levels of BV/TV, BMD, Tb.Th, Tb.N, and Tb.Sp. Specifically, the BV/TV index increased from 8.67 to 11 %, the BMD level increased from 0.11 to 0.123 g/mm^3^, and the Tb.Th level increased from 0.01 5 to 0.025 mm, Tb.N level increased from 1.897 to 2.277 mm, and Tb.Sp level decreased from 0.917 to 0.84 mm. In contrast, following long-duration swimming training, there was a more substantial enhancement in BV/TV, BMD, Tb.Th, Tb.N, and Tb.Sp levels compared to the ovariectomized group. Specifically, the percentage of BV/TV exhibited a significant increase of 14.67 %, while the BMD level increased to 0.203 g/mm^3^, the Tb.Th level increased to 0.047 mm, the Tb.N level increased to 4.6 mm, and the Tb.Sp level decreased to 0.687 mm. These findings were statistically significant (p<0.05; Figures 2 and 3).

### Effect of swimming on the bone resorption markers CTX-Ⅰand TRAP-5b in ovariectomized osteoporotic rats

**Figure 4:**
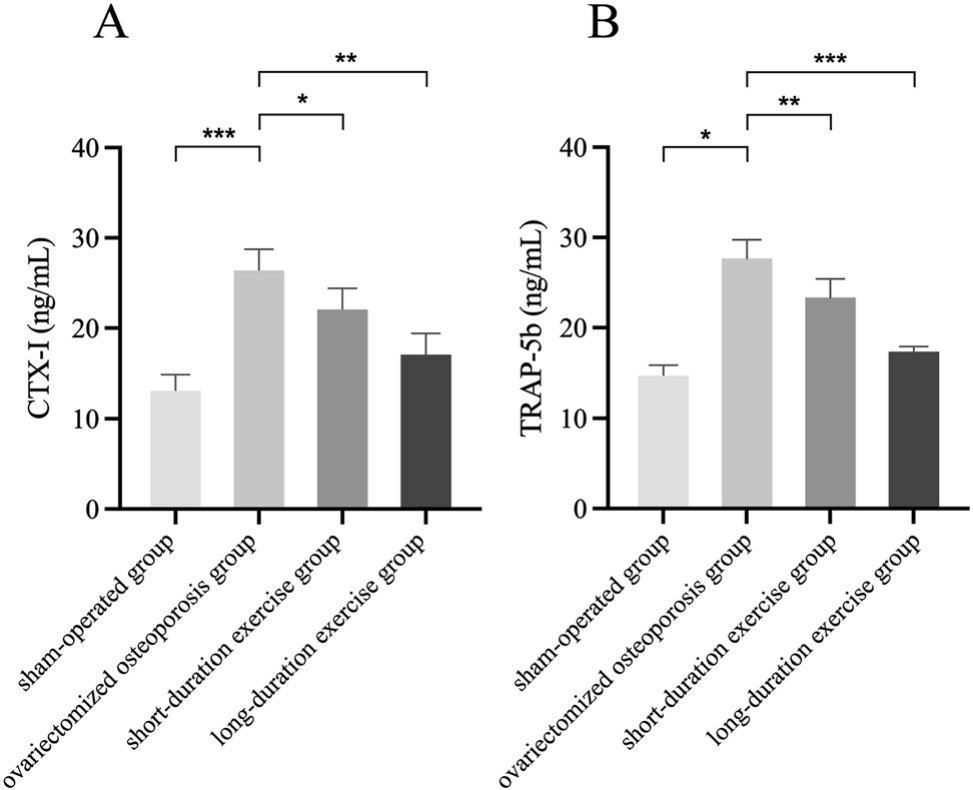
Detection of bone resorption marker CTX-Ⅰand TRAP-5b. Compared to the ovariectomized osteoporotic group, *p<0.05, **p<0.01, ***p<0.001.

CTX-I and TRAP-5b have been identified as markers of bone resorption. Levels of these bone resorption markers increased in the ovariectomized osteoporosis group of rats compared with the sham-operated group. The increase was statistically significant (p<0.05). Following short-duration swimming training, a decline in CTX-I and TRAP-5b levels was observed in the ovariectomized osteoporosis group when compared with the sham-operated group. Specifically, the CTX-I level exhibited a decline from 26.67 to 22.33 ng/mL, while the TRAP-5b level diminished from 27.67 to 23.33 ng/mL. However, it is noteworthy that the enhancement in CTX-I and TRAP-5b levels was more pronounced following long-duration swimming training. Notably, both CTX-I and TRAP-5b levels exhibited a decline to 17.33 ng/mL, a difference that was statistically significant (p<0.05; Figure 4).

### Effect of swimming on the bone formation markers BGP and BALP in ovariectomized osteoporotic rats

**Figure 5:**
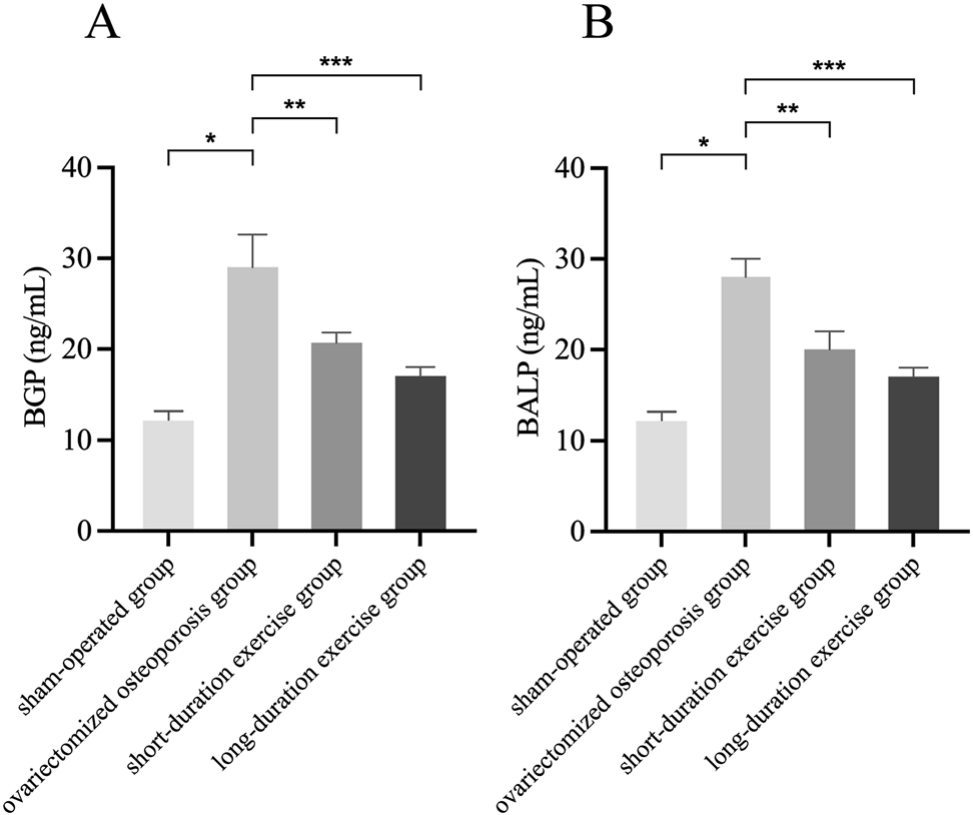
Detection of bone resorption marker BGP and BALP. Compared to the ovariectomized osteoporotic group, *p<0.05, **p<0.01, ***p<0.001.

Bone formation markers, BGP and BALP, exhibited significantly higher levels in the ovariectomized osteoporotic rat group compared to the sham-operated group (p<0.05). The levels of BGP (from 11.67 to 29 ng/mL) and BALP (from 11.677 to 28 ng/mL) increased in a statistically significant manner in the ovariectomized osteoporotic group compared to the sham-operated group. Subsequent to short-duration swimming training, a decline in BGP level to 20.67 ng/mL and BALP level to 20 ng/mL was observed. Conversely, following long-duration swimming training, a more pronounced enhancement in BGP and BALP levels was evident, with both decreasing to 17 ng/mL, yielding statistically significant differences (p<0.05; Figure 5).

### Effect of swimming on the expression of OPG, RANKL, and RANK mRNA in ovariectomized osteoporotic rats

**Figure 6:**
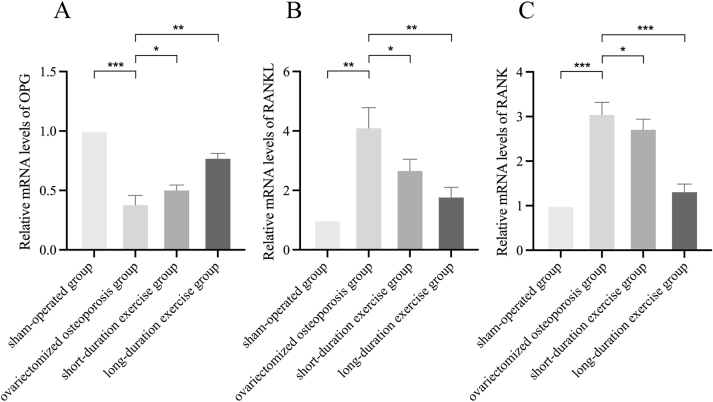
Relative mRNA expression levels of OPG (A), RANKL (B), and RANK (C). Compared to the ovariectomized osteoporotic group, *p<0.05, **p<0.01, ***p<0.001.

The relative expression of OPG (from 1 to 0.387) mRNA was decreased and the relative expression of RANKL (from 1 to 4.13) and RANK (from 1 to 3.07) mRNA was increased in the ovariectomized osteoporosis group compared to the sham-operated group, and the differences were statistically significant (p<0.05). After exercise treatment, compared with the ovariectomized osteoporosis group, the relative expression of OPG mRNA increased to 0.51, the relative expression of RANKL mRNA decreased to 2.7, and the relative expression of RANK mRNA decreased to 3.07. The relative mRNA expression of OPG increased to 0.777, RANKL and RANK decreased to 1.8 and 1.33, respectively (p<0.05; Figure 6; Table 1).

**Table 1:** Primer sequences for qPCR used in this study.

Gene	Accession#	Forward primer	Reverse primer
OPG	NM_012870.2	AGA​CGT​CAT​CGA​AAG​CAC​CC	GCA​CAG​GGT​GAC​ATC​TAT​TCC​A
RANK	NM_001271235.1	GAG​CTC​AAC​ATC​CCT​TGC​AG	TCC​CTT​GGT​GTG​CTT​CCA​TC
RANKL	NM_057149.2	GTA​CTT​TCG​AGC​GCA​GAT​GGA	GTC​GAG​TCC​TGC​AAA​CCT​GTA

### Effect of swimming on the expression of OPG, RANKL, and RANK proteins in ovariectomized osteoporotic rats

**Figure 7:**
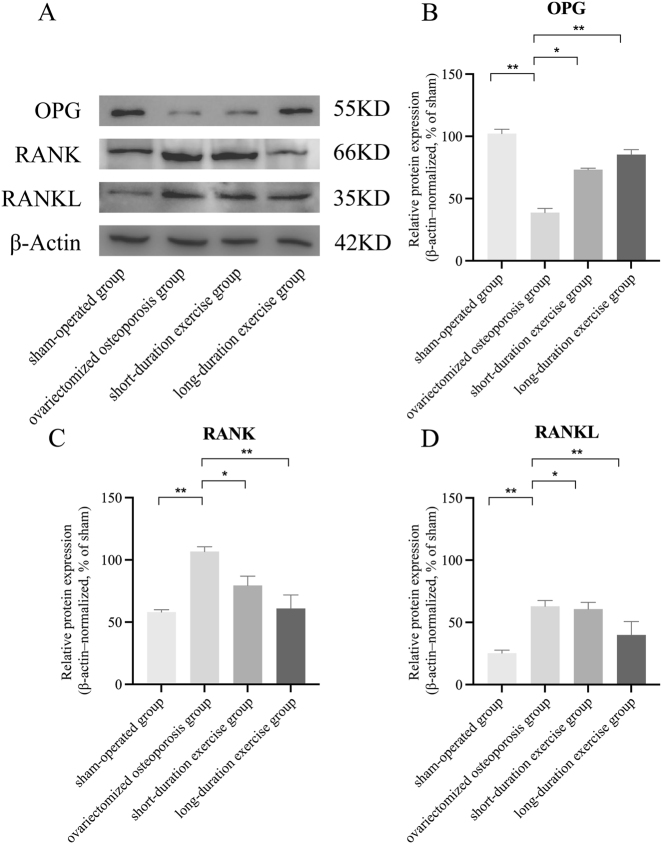
Effect of swimming on the expression of OPG, RANKL, and RANK in ovariectomized osteoporotic rats. Western blot bands were examined in triplicate and quantified by densitometry. Band intensities were normalized to β-actin (loading control) and expressed as % of the sham-operated group (set to 100 %) for quantitative analysis. Compared with the OVX osteoporotic group, *p<0.05, **p<0.01.

Comparing the ovariectomized osteoporosis group with the sham-operated group, OPG protein expression was significantly decreased from 102.23 to 38.68 %, RANKL protein expression was increased from 25.26 to 62.97 %, and RANK protein expression was increased from 58.04 to 106.71 %, and the differences were statistically significant (p<0.05). After swimming treatment, OPG protein expression increased to 73.18 %, and RANKL and RANK protein expression decreased to 60.66 % and 79.40 %, respectively, in the short-duration swimming training group compared with the ovariectomized osteoporotic group. After long-duration swim training, the improvement was more significant compared with the short-duration swim training group. Among them, OPG protein expression was increased to 85.25 %, and protein expression of RANK and LRANK was decreased to 39.89 % and 60.90 %, with statistically significant differences (p<0.05; Figure 7).

## Discussion

In the present study, we found that swimming training alleviated ovariectomy-induced bone loss and improved trabecular microarchitecture, accompanied by reduced bone resorption markers (CTX-I and TRAP-5b). These effects were also accompanied by increased OPG and decreased RANKL and RANK expression, suggesting a potential involvement of the OPG/RANKL/RANK axis in swimming-associated skeletal protection.

Osteoporosis is a global health problem affecting millions of men and women around the world, and is particularly prevalent in older people and postmenopausal women. The aging process accelerates bone loss, especially in postmenopausal women. Estrogen (E) deficiency due to ovarian dysfunction is the main reason for the development of postmenopausal osteoporosis [33], 34]. The sharp decline in oestrogen levels leads to a dramatic increase in the incidence of osteoporosis and the risk of fractures, which can severely reduce quality of life and even increase mortality. PMO can be treated with drugs (e.g. bisphosphonates, dinosemide, teriparatide, etc.), hormones and lifestyle changes, but there are challenges with compliance, side effects and personalised treatment. Among these, hormone replacement therapy (HRT) has been widely used in recent decades [33], 34]. However, prolonged stimulation of the endometrium by estrogen can cause hyperplasia, which can lead to endometrial cancer. Prolonged exposure of the breast to estrogen can also lead to the development of breast cancer. Therefore, it is imperative to find a more favorable approach for postmenopausal women with osteoporosis as a priority.

Exercise has an anabolic effect on bone either directly through mechanical signals produced by muscle contraction or indirectly through endocrine control and is the preferred method of treatment for obesity-related poor bone health [35], [36], [37]. Consequently, bone metabolism shows how closely muscle and bone interact [38]. Exercises that increase bone metabolism include weight-bearing activities like walking, running, and weight training; non-weight-bearing activities like swimming and cycling [39]. Yet, current research indicates that non-weight-bearing activities like swimming are equally useful for enhancing bone metabolism [29], [40], [41], [42]. Osteoporosis leads to decreased BMD, reduced (BV/TV), and degradation of trabecular bone structure, which in turn reduces bone strength and stability [43]. Accelerated bone resorption and slowed bone formation decrease bone mineralization, and these changes increase the risk of fracture [44]. Consequently, swimming, a low-impact aerobic exercise, may help to counteract the loss of bone density and the development of osteoporosis by improving blood circulation, promoting bone formation, and enhancing the tolerance of bones to mechanical loads [15]. In particular, Falcai et al. demonstrated that three weeks of swimming increased BMD and microstructure in rats with reduced bone density by hindlimb suspension as effectively as jumping exercises [21]. In addition, Ju et al. showed that swimming exercise significantly inhibited the loss of bone mineral density and the deterioration of trabecular bone microarchitecture in ovulated rats, suggesting that swimming has a potentially beneficial effect in ameliorating osteoporosis [45]. These findings imply that bone health can be improved and maintained by muscular contraction alone, without the addition of weight. Gene expression or different hormones secreted in the bone or skeletal muscle tissues control the mechanisms that promote bone metabolism by muscular contraction [46]. The monitoring of markers such as BMD and bone trabeculae is instrumental in evaluating the severity of osteoporosis and the state of bone health. Consequently, in the present study, following the implementation of the swimming treatment, an examination of the osteoporosis markers was conducted for the purpose of testing. The results indicated that the levels of the osteoporosis markers BV/TV, BMD, Tb.Th and Tb.N were increased and levels of Tb.Sp were decreased compared to the ovariectomized osteoporosis group; the number of bone trabeculae was significantly increased and the trabeculae were more tightly connected; levels of the bone resorption markers CTX-I and TRAP-5b were decreased; and levels of the bone formation markers BGP and BALP were decreased in the rats. Significant increases in BV/TV, BMD, Tb.Th, and Tb.N indicate that swimming enhances trabecular structure, increases BMD, and augments the number of trabeculae, thereby corroborating its mitigating effect on osteoporosis. Tb.Sp was reduced compared to the ovariectomized osteoporotic group, suggesting tighter connections of trabeculae and repair of bone structure. The observed decrease in the level of CTX-I and TRAP-5b further corroborates the efficacy of swimming in decelerating the progression of bone loss. Reduced levels of BGP and BALP suggest that swimming training may inhibit excessive bone resorption and bone formation activity, helping to restore bone metabolic balance and slow the progression of osteoporosis. Consequently, these findings collectively indicate that swimming exerts a substantial inhibitory effect on bone loss in ovariectomized osteoporotic rats and mitigates the pathological manifestations of osteoporosis by enhancing trabecular structure, diminishing bone resorption, and fostering bone formation.

In addition, the present study focused on the finding that the relative expression of OPG mRNA and protein was elevated and the relative expression of RANKL and RANK mRNA and protein were decreased in the swimming-treated group compared with the ovariectomized osteoporosis group. Current research has identified the OPG/RANKL/RANK signaling pathway as an important pathway regulating bone metabolism, mainly by influencing the differentiation and maturation of osteoclasts, thus affecting the process of bone metabolism [25]. Among them, RANKL is a signalling molecule that promotes the formation of osteoclasts, and the binding of RANKL to RANK promotes the differentiation and activity of osteoclasts. OPG, a soluble receptor secreted by osteoblasts, is a key regulator of bone resorption by competitively binding to RANKL and blocking the pathway linking RANKL to RANK, at which point the level or activity of OPG increases and inhibits osteoclast activation and differentiation of osteoclasts, thereby preventing the initiation of subsequent signaling pathways and ultimately regulating the activity of mature osteoclasts and the rate of apoptosis, thereby attenuating the effects of bone resorption. The balance of the OPG/RANKL/RANK pathway is essential for maintaining bone mineral density and bone health. When the ratio of RANKL to OPG is increased, osteoclast activity is enhanced, leading to increased bone resorption, which plays a key role in the development of bone metabolic disorders such as osteoporosis. The OPG/RANKL/RANK signaling pathway, as a downstream signaling pathway of the estrogen receptor activation pathway, is particularly important in postmenopausal osteoporosis [47]. Estrogen, through its receptor, affects the expression of OPG and RANKL, which in turn regulate bone metabolism. When estrogen levels decrease, such as in postmenopausal women, RANKL expression can increase while OPG levels decrease, leading to increased osteoclast activity and increased bone resorption [48]. Furthermore, a study by Nair S et al. revealed that OPG rs2073618 and rs3102735 polymorphisms were associated with spinal BMD, suggesting their potential as diagnostic markers for identifying women with low BMD [49]. A similar finding was reported in a study by Peng et al. where the A163G, G1181C, and T950C polymorphisms of the OPG gene were found to be associated with BMD in postmenopausal women [50]. This suggests that these polymorphisms of the OPG gene may affect BMD, particularly in postmenopausal women. In light of the extant studies and the findings of the present experiment, it can be hypothesized that swimming may have been associated with changes in the OPG/RANKL/RANK pathway by increasing OPG expression and decreasing RANKL expression. These changes may have been associated with the process of bone resorption, improvements the BMD and trabecular structure, and attenuation the symptoms of postmenopausal osteoporosis. This finding not only may provide a new perspective for understanding how exercise swimming affects bone metabolism, but also may provide a preliminary scientific basis for the development of non-pharmacological interventions, highlighting the potential importance of appropriate exercise swimming in the prevention and treatment of postmenopausal osteoporosis.

To enhance the translational relevance of the present findings, it is important to consider whether the swimming-associated effects observed in ovariectomized rats are comparable to those in postmenopausal women. The OVX rat is a widely used preclinical model of estrogen deficiency–related bone loss and recapitulates key features of postmenopausal osteoporosis; thus, our results may provide mechanistic clues that are potentially relevant to the clinical setting. Notably, evidence from postmenopausal women indicates that aquatic exercise programs can lead to favorable changes in bone-related outcomes, including improvements in hip and spine areal bone density and beneficial shifts in bone turnover markers, supporting aquatic exercise as a feasible non-pharmacological strategy in this population [23]. Nevertheless, direct extrapolation from OVX rats to humans should be made cautiously, because the rates and dynamics of bone remodeling, as well as the magnitude of exercise responsiveness, may differ substantially between species. Moreover, systemic metabolic profiles – including gut microbiota–related metabolites such as trimethylamine N-oxide (TMAO) – may not be directly comparable between animal models and humans, and such differences could influence bone remodeling and contribute to variability in observed effects. Therefore, further translational studies, ideally incorporating clinical validation in postmenopausal women together with parallel biomarker assessments, are warranted to better define the comparability and extrapolative validity of swimming-associated skeletal benefits.

Nevertheless, several limitations should be acknowledged. Although the OPG/RANKL/RANK pathway was assessed at the mRNA and protein levels, these expression-based findings are insufficient to establish a causal link between pathway modulation and swimming-induced protection against bone loss, as functional validation (e.g., pharmacological inhibition/neutralization of RANKL, blockade of OPG, or genetic perturbation of pathway components) was not performed. In addition, the current study did not include a comparator group such as a weight-bearing exercise intervention or an anti-osteoporotic drug control (e.g., alendronate); therefore, the relative efficacy of duration-based swimming cannot be directly benchmarked against established exercise modalities or pharmacological therapies. Future studies incorporating mechanistic perturbations together with appropriate reference groups are warranted to clarify causality and better delineate the unique and comparative effects of swimming on bone metabolism.

## Conclusions

In conclusion, swimming alleviated bone loss and was accompanied by changes in the OPG/RANKL/RANK signaling pathway in ovariectomized osteoporotic rats, suggesting a potential non-pharmacological adjunct option for the management of postmenopausal osteoporosis.

## Supplementary Material

Supplementary Material
