# Supplementary material for: Effects of swimming on bone loss and mechanisms in ovariectomized osteoporotic rats
Source: Open Med (Wars). 2026 Feb 23;21(1):20261382. doi: 10.1515/med-2026-1382 (PMC12922758; doi:10.1515/med-2026-1382)
Supplement: Supplementary file 1 — Supplementary Material [file j_med-2026-1382_suppl_001.doc]

**Supporting Information**

**Effects of Swimming on Bone Loss and Mechanisms in Ovariectomized Osteoporotic Rats**

Yingying Zhao^1#^, Yinyu Chen^2#^, Xinyan Yang^2#^, Yilin Wang^3^, Yangyang Zhang^1^, Lin Li^4*^, Peng Zhang^2*^

^1^ Department of Physical Education, Hainan Medical University, Haikou 571199, China.

^2^ Key Laboratory of Tropical Translational Medicine of Ministry of Education, College of Basic Medicine, Hainan Medical University, Haikou 571199, China

^3^ UWE College of Hainan Medical University, Haikou 571199, China

^4^ College of Physical Education, Huanghe S & T University, Zhengzhou 450000, China

^#^ These authors contributed equally to this work.

**^*^Correspondence:**

Lin Li, College of Physical Education, Huanghe S & T University, Zijingshan Road 666#, Zhengzhou, China. E-mail: 49015089@qq.com.

Peng Zhang, Key Laboratory of Tropical Translational Medicine of Ministry of Education, College of Basic Medicine, Department of Forensic Medicine, Hainan Provincial Academician Workstation (tropical forensic medicine), Hainan Provincial Tropical Forensic Engineering Research Center, Hainan Medical University, Xueyuan Road 3#, Longhuaqu, Haikou, China. E-mail: 972421821@qq.com.

**
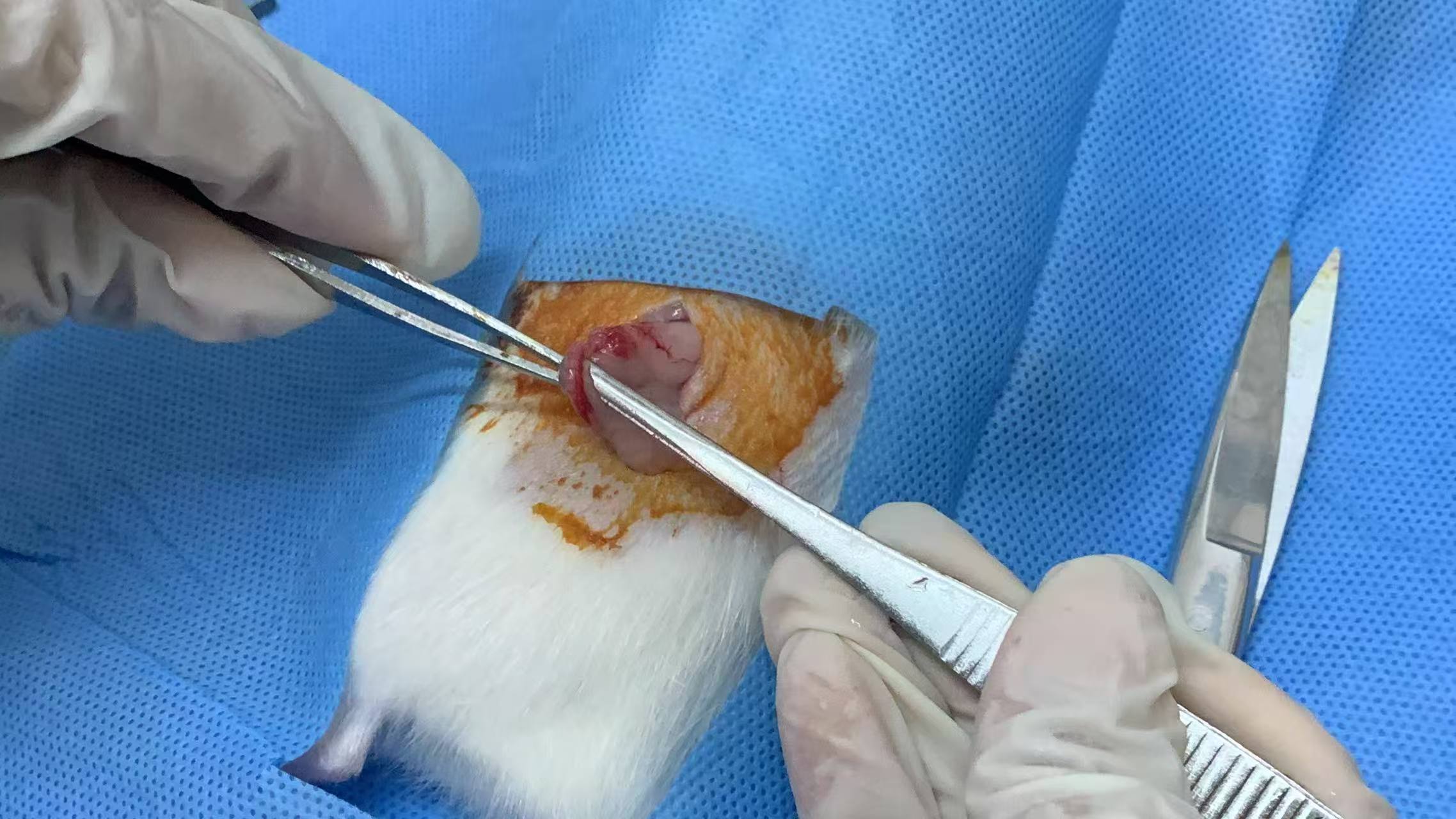
**

**
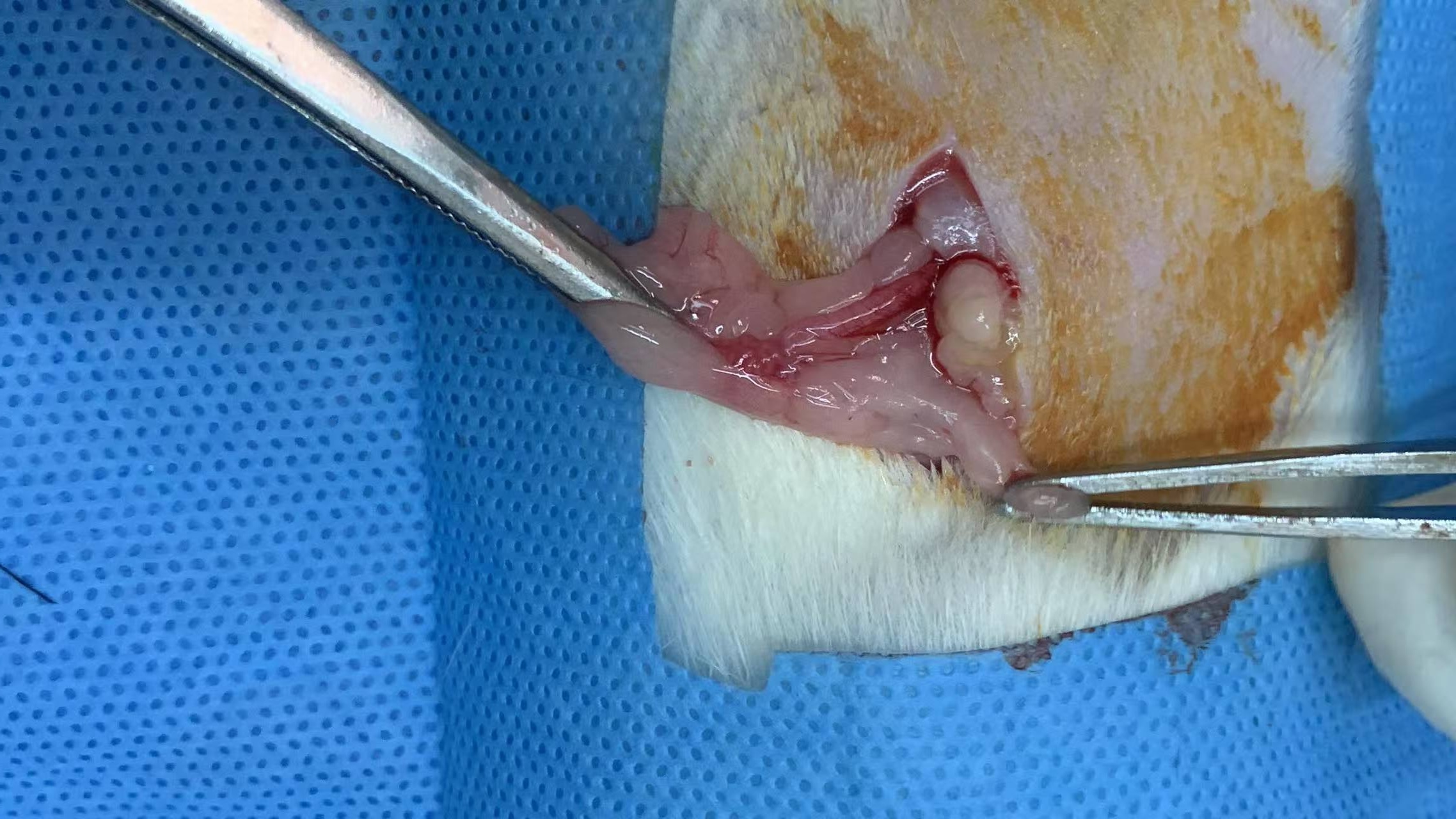
**


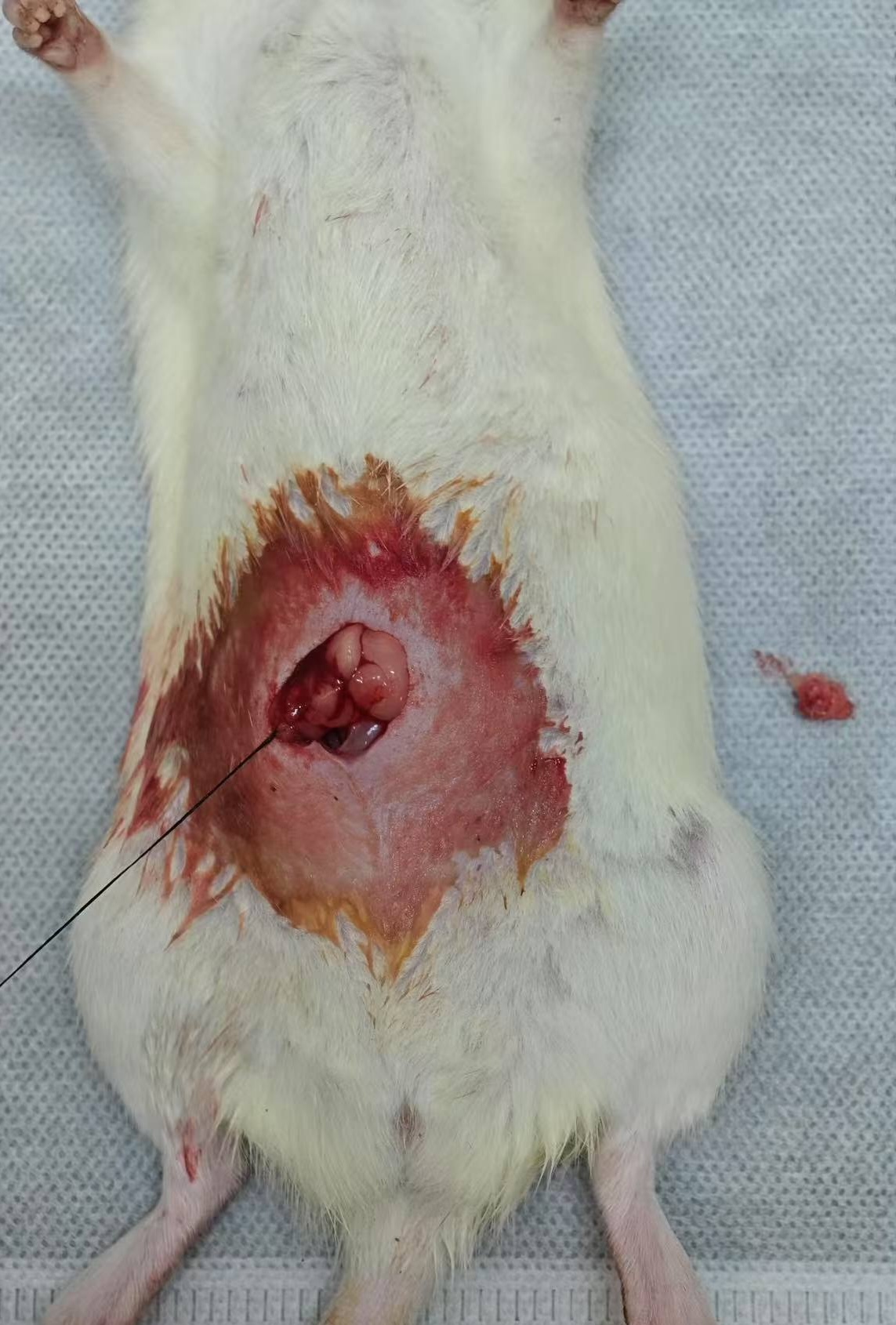

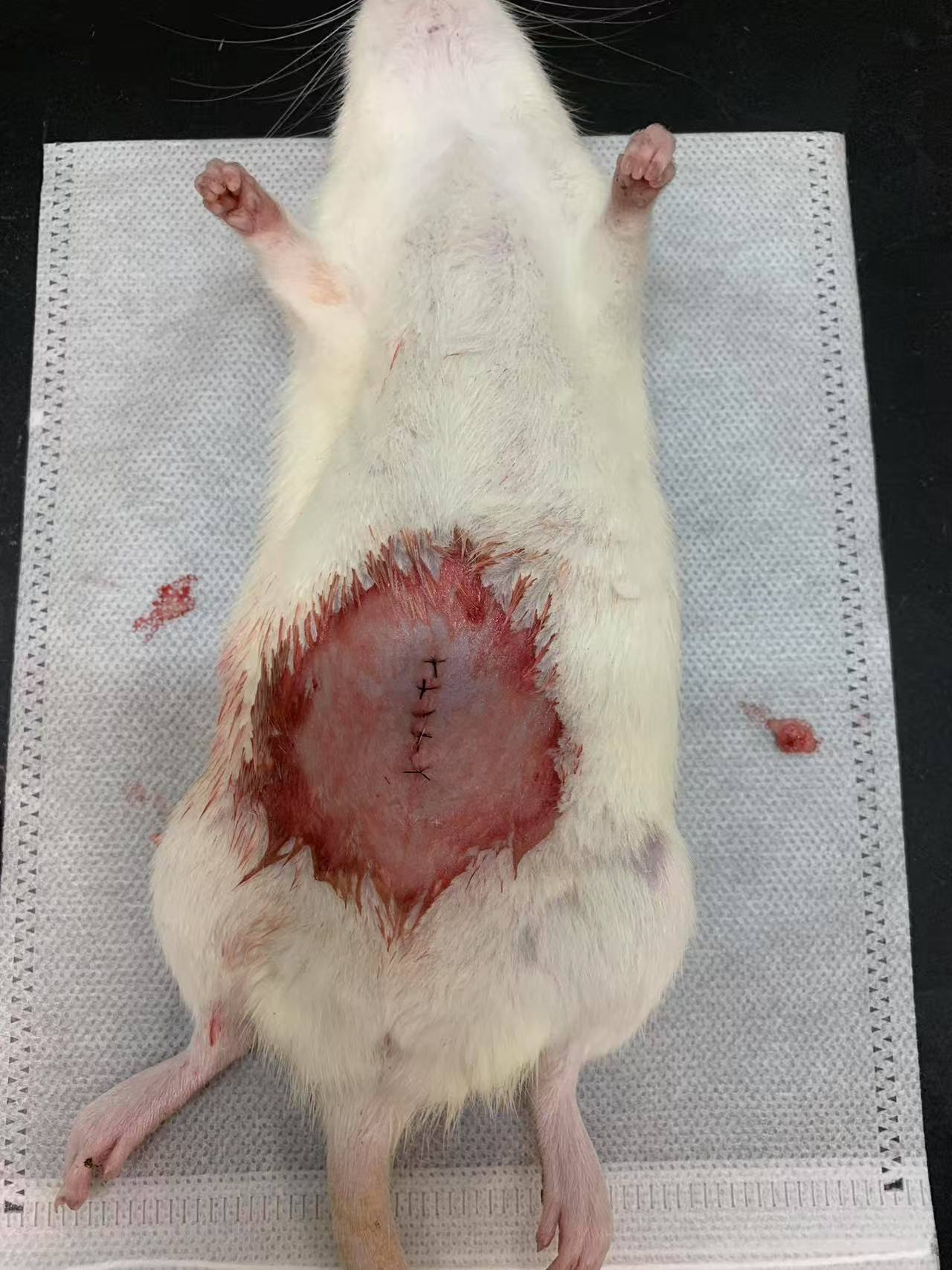


OVX model images
